# Enhancing the Mechanical Properties of Sulfur-Modified Fly Ash/Metakaolin Geopolymers with Polypropylene Fibers

**DOI:** 10.3390/polym17152119

**Published:** 2025-07-31

**Authors:** Sergey A. Stel’makh, Evgenii M. Shcherban’, Alexey N. Beskopylny, Levon R. Mailyan, Alexandr A. Shilov, Irina Razveeva, Samson Oganesyan, Anastasia Pogrebnyak, Andrei Chernil’nik, Diana Elshaeva

**Affiliations:** 1 Department of Unique Buildings and Constructions Engineering, Don State Technical University, 344003 Rostov-on-Don, Russia; sergej.stelmax@mail.ru (S.A.S.); lrm@aaanet.ru (L.R.M.); alexandr_shilov@inbox.ru (A.A.S.); razveevai@mail.ru (I.R.); alfateh@list.ru (S.O.); afedchishena@mail.ru (A.P.); chernila_a@mail.ru (A.C.); diana.elshaeva@yandex.ru (D.E.); 2 Department of Engineering Geometry and Computer Graphics, Don State Technical University, 344003 Rostov-on-Don, Russia; au-geen@mail.ru; 3 Department of Transport Systems, Faculty of Roads and Transport Systems, Don State Technical University, 344003 Rostov-on-Don, Russia

**Keywords:** technical sulfur (TS), geopolymer composite, strength, dispersed reinforcement, polypropylene fiber (PF), structure

## Abstract

High demand for sustainable solutions in the construction industry determines the significant relevance of developing new eco-friendly composites with a reduced carbon impact on the environment. The main aim of this study is to investigate the possibility and efficiency of using technical sulfur (TS) as a modifying additive for geopolymer composites and to select the optimal content of polypropylene fiber (PF). To assess the potential of TS, experimental samples of geopolymer solutions based on metakaolin and fly ash were prepared. The TS content varied from 0% to 9% by weight of binder in 3% increments. In the first stage, the density, compressive and flexural strength, capillary water absorption and microstructure of hardened geopolymer composites were tested. The TS additive in an amount of 3% was the most effective and provided an increase in compressive strength by 12.6%, flexural strength by 12.8% and a decrease in capillary water absorption by 18.2%. At the second stage, the optimal PF content was selected, which was 0.75%. The maximum increases in strength properties were recorded for the composition with 3% TS and 0.75% PF: 8% for compression and 32.6% for bending. Capillary water absorption decreased by 12.9%. The geopolymer composition developed in this work, modified with TP and PF, has sufficient mechanical and physical properties and can be considered for further study in order to determine its competitiveness with cement composites in real construction practice.

## 1. Introduction

Technical sulfur is a by-product of gas processing and oil refineries. Every year, the global amount of sulfur produced is growing. The world leaders in sulfur production are China—up to 19 million tons per year—the United States—up to 8.6 million tons—Russia—up to 7 million tons—Saudi Arabia—up to 8 million tons—and Canada—up to 4.9 million tons [1,2]. In this regard, the search for various alternative options for the use of technical sulfur, aimed at preventing its accumulation and complete utilization, is of particular relevance [3,4]. The topic of using various types of waste in the construction industry, including the manufacture of new types of building materials, in particular composites, is highly relevant and is actively developing both in our country and abroad [5,6]. Sulfur as a by-product can be used to manufacture new types of building materials. Technical sulfur has found its most popular application in the technology of bitumen binders, where sulfur acts as a modifying additive or filler. Introducing sulfur into the composition of highly concentrated bitumen with a high content of tire rubber made it possible to increase its resistance to aging and improve elasticity [7]. When the sulfur concentration in bitumen is higher than 20% (by weight of binder), some of its crystalline phase acts as a filler, which subsequently has a positive effect on the properties of asphalt concrete mixtures and increases their rigidity [8]. Modification of bitumen with organic sulfur polymers makes them less sensitive to high temperatures and more resistant to constant deformation and aging processes [9]. Sulfur dissolved in bitumen improves its low-temperature properties and resistance to fatigue cracking [10]. The combination of phenol-rich compounds and sulfur makes it possible to obtain a more environmentally friendly bitumen with optimal properties [11,12,13]. Sulfur is used as a binder in the production of an artificial composite material—sulfur concrete. An effective composition of sulfur concrete was selected, where styrene and bitumen were used as modifiers [14]. The sulfur concrete composite presented in the work [15] had optimal physical and mechanical properties and high chemical resistance, studied by keeping samples in a solution of sulfuric acid and sulfate. Sulfur concrete on artificial fine aggregate has high chemical resistance [16]. Another advantage of sulfur concrete is that any types of coarse and fine aggregate can be used in it, since this is a water-repellent concrete. Modified sulfur concrete mixtures can be used, along with cement concrete mixtures, for the production of both small-piece factory-made products such as interlocking joints (paving slabs) and large-sized products (foundation blocks) [17].

Besides cement or sulfur concretes, geopolymer concrete currently remains a popular and environmentally friendly building material. Various types of industrial waste are used to produce geopolymer composites. Many scientific studies are aimed at developing effective geopolymer concretes that combine several types of waste [18,19,20,21,22,23,24,25]. An environmentally friendly composition of self-compacting geopolymer concrete was obtained from a mixture of fly ash, slag and basalt fiber, and compressive strength up to 34 MPa [26]. The combination of ash and slag as aluminosilicate binders and steel and polypropylene fibers made it possible to obtain a composite with improved compressive strength, splitting, bending, and impact toughness [27]. A study on the optimization of fly ash (FA), ground granulated blast furnace slag (GGBS) and silica (PS) as a partial replacement for traditional cementitious materials allowed us to select the most optimal combination of these components in the ratio of 50% FA, 40% GGBS and 10% PS (by weight of binder), providing high strength properties [28]. Geopolymer concrete based on FA, coal shale, nanosilica and carbon fiber had good resistance to freezing and thawing and abrasion and was recommended for use in road infrastructure [29]. Composite sandwich panels containing nylon fiber-reinforced geopolymer showed improved performance properties under bending load [30]. A popular aluminosilicate component for the production of geopolymer concrete is metakaolin. Geopolymer based on metakaolin demonstrated compressive strength up to 79.4 MPa and good resistance to aggressive acidic environments [31]. The optimal mechanical strength and reduced drying shrinkage of metakaolin-based geopolymer composite were observed when it contained 10% cement (by binder weight) [32]. An important aspect in the manufacture of geopolymers is the ratio of silicon and aluminum, with an optimal range of 1.5–1.9, which is proven by a study that studied the ratio of these components [33]. Geopolymer solutions based on metakaolin and sewage sludge ash demonstrated efficiency with strength levels of 42.5 MPa and 33.7 MPa, respectively [34].

Using various types of fiber in the development of geopolymer mortar and concrete compositions is a popular formulation solution and allows for additional improvement of the properties of composites [35,36]. Basalt and polypropylene fibers seem to be the most popular. Basalt fibers work well in the structure of geopolymer solutions. The introduction of 1.2% basalt fiber increased the compressive strength by up to 20% [37]. Columns composed of geopolymer concrete with basalt fiber showed higher ultimate load and ductility [38]. The inclusion of 1.5% polypropylene fibers in the composition of the geopolymer solution made it possible to significantly increase its flexural strength and flexural viscosity index [39]. Self-healing geopolymer solutions with 0.75% polypropylene fibers showed the best compressive strength [40]. A number of studies have also proven the possibility of effectively using polyvinyl alcohol, steel, jute and sisal fibers [41,42,43,44]. The introduction of environmentally friendly materials into the modern construction industry is one of the most important steps towards a low-carbon future [45,46]. Geopolymer concrete, where various types of industrial waste are used as binders, is one of the modern types of environmentally friendly building materials [47,48,49]. Thus, this study, aimed at developing new sustainable types of geopolymer composites using fly ash (FA) and technical sulfur (TS) waste, is highly relevant. In the modern scientific literature on geopolymer composites with oil refining waste in the form of TS, a significant scientific deficit has been identified. There are quite a few works devoted to this topic [50]. The scientific novelty of the work consisted in assessing the possibility of using TS as a modifying additive for the manufacture of geopolymer composites and obtaining new dependencies of the properties and structure of a geopolymer composite on a combined binder in the form of metakaolin and fly ash, modified with technical sulfur and dispersed-reinforced with polypropylene fiber, on the recipe parameters of the mixture. The objective of this study was to determine the optimal effective amount of TS for modifying geopolymer composites based on metakaolin and fly ash and the percentage of polypropylene fiber reinforcement to obtain a new type of environmentally friendly geopolymer composite.

The objectives of the work included:

- selection of a geopolymer formulation on a mixed binder in the form of metakaolin and fly ash with different dosages of technical sulfur and polypropylene fiber;
- production of experimental test samples of geopolymer and determination of their density, compressive and flexural strength and capillary water absorption;
- study of the structure of geopolymers modified with TS, using SEM and EDX analysis;
- analysis of the experimental data results and selection of the most optimal ranges of the modifying additive content of technical sulfur and polypropylene fiber, ensuring the production of geopolymer composites with the required performance properties;
- assessment of the possible area of application of a geopolymer composite modified with technical sulfur in real construction practice.

## 2. Materials and Methods

### 2.1. Materials

The following were used as the main raw materials for the preparation of geopolymer solutions: metakaolin (M) (SINERGO Group, Zheltinsky, Russia), fly ash (FA) (Novocherkassk State District Power Plant, Novocherkassk, Russia), potassium liquid glass (K_2_O(SiO_2_)n) (Promsteklocenter, Novomoskovsk, Russia) and quartz sand (QS) (Nedra, Samarskoye, Russia). Technical sulfur (TS) (Novoshakhtinsk Oil Refinery, Novoshakhtinsk, Russia), which is a by-product of oil refining, was used as a modifying additive. For dispersed reinforcement, polypropylene fiber (PF) (Armplast, Taganrog, Russia) was used.

The main properties of the raw materials are presented in Table 1.

**Figure 1:**
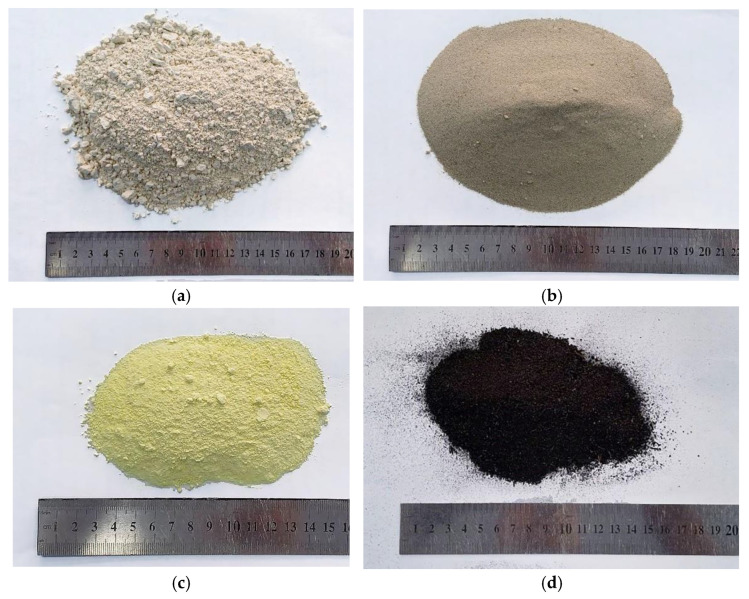
Appearance of raw materials: (**a**) metakaolin; (**b**) sand; (**c**) sulfur; (**d**) fly ash.

The appearance of the raw materials is shown in Figure 1.

### 2.2. Methods

The production of geopolymer solutions was carried out in accordance with the recipe presented in Table 2.

**Figure 2:**
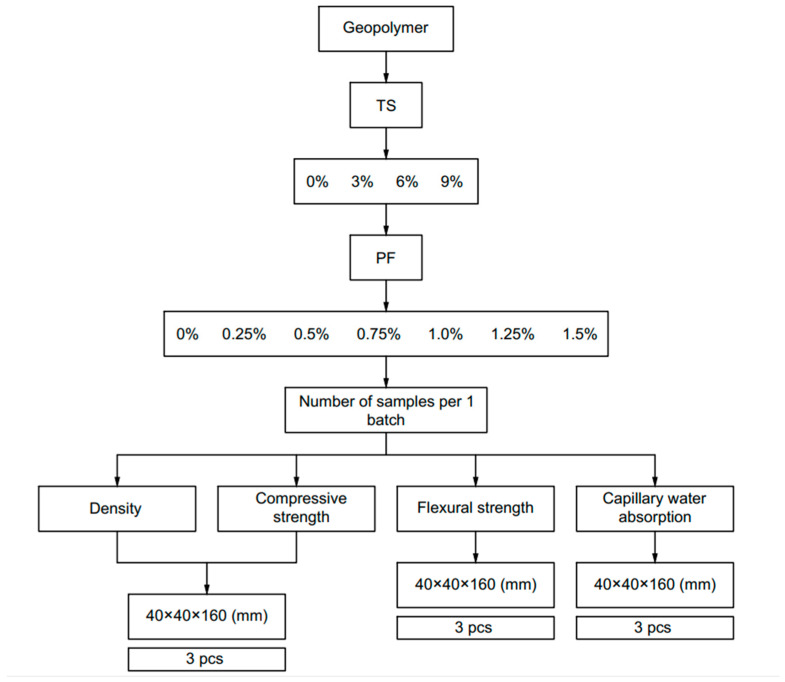
Experimental research program.

The selected formulations with established dosages were determined on the basis of a number of trial experiments. Geopolymer solutions were prepared in laboratory conditions in accordance with the recipe presented in Table 2. Aluminosilicate components were weighed and mixed dry. Then, an alkaline activator with sulfur dissolved in it was poured into the M + FA mixture and mixed until a homogeneous state was obtained. Sulfur was dissolved in the alkaline activator by stirring in a mixer according to [50]. PF was added manually at the very end, and the geopolymer solution was mixed again. The fiber was divided into approximately three equal doses, each of which was evenly distributed over the entire surface of the mixture and mixed for 30 s. This operation was repeated and ensured the most uniform distribution of fiber over the entire volume of the mixture. The homogeneity of the fiber distribution was controlled on samples of fresh geopolymer mixture and hardened samples after strength testing visually and using an MBS-10 optical microscope (Izmeritelnaya Tekhnika, Moscow, Russia) [48]. After production, the geopolymer solution was poured into 40 × 40 × 160 mm molds and compacted for 60 s on a laboratory vibration platform. The finished samples were covered with film and stored in the molds for 3 days. The first 3 days are a critical period for geopolymer concrete, when initial setting and hardening occur. Curing in forms helps prevent premature drying, cracking and deformation of samples. Preventing deformations in the initial period ensures the accuracy of strength test results. Then, the molds were opened, and the samples were removed and hardened in laboratory conditions (temperature 20 ± 2 °C, relative air humidity 95 ± 5%) for another 25 days. The geopolymer samples were tested in accordance with the program presented in Figure 2.

A total of 198 prism samples were manufactured (9 for each type of mixture). The amount of sulfur and fiber is presented as a percentage of weight of binder.

The density of the hardened geopolymer composites was determined at the age of 28 days in accordance with the requirements of [51] and was calculated using the formula:

(1) $$\rho =\frac{m}{V}\;\times \;1000$$

where *m* is the mass of the sample (g); *V* is the volume (cm^3^).

**Figure 3:**
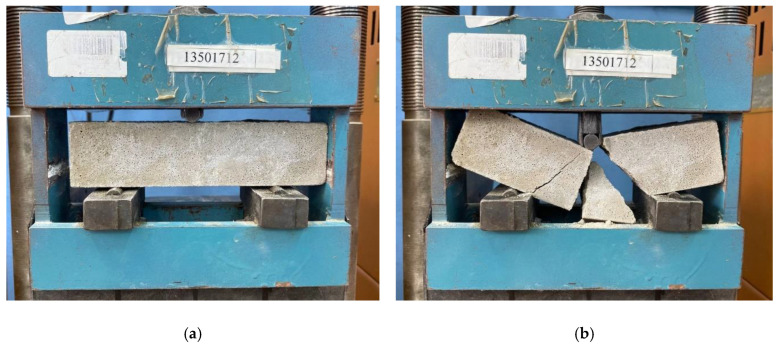

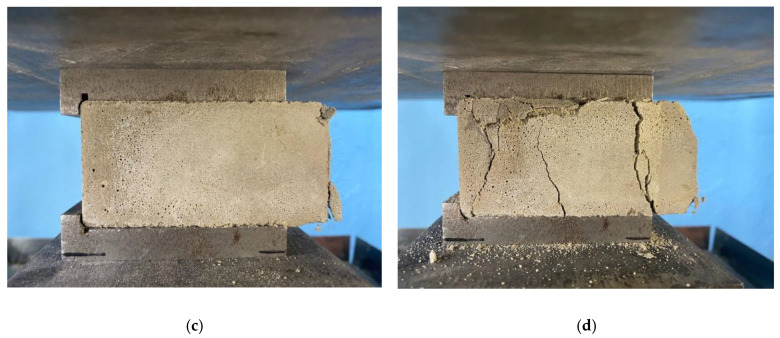
Determination of flexural strength: (**a**) before failure, (**b**) after failure, and compressive strength: (**c**) before destruction, (**d**) after destruction of geopolymer mortar samples.

The compressive and flexural strength of geopolymer composites was determined in accordance with the requirements of [52]. Before testing, all samples were visually inspected for defects in the form of cracks, rib fractures, cavities, delaminations and insufficient compaction. Samples with these types of defects on the surface with a length of more than 3 mm and a depth of more than 2 mm, as well as samples with traces of delamination and insufficient compaction, were not allowed to be tested. Then the geometric parameters of the samples were measured. The samples were installed in a special laboratory setup and loaded at a load increase rate of 0.05 ± 0.01 MPa/s. Then the halves of the prism samples were placed in special pressure plates and tested for compression at a load increase rate of 0.6 ± 0.2 MPa/s (Figure 3). Compressive (*R*) and bending ($R_{tb}$) strengths were calculated using Formulas (2) and (3):

(2) $$R=\frac{F}{S}$$

where *F* is the breaking load (N); *S* is the working cross-sectional area of the sample (mm^2^).

(3) $$R_{tb}=0.00234\times F$$

The capillary water absorption of geopolymers was determined in accordance with the requirements of [52]. Samples measuring 40 × 40 × 160 mm, dried to a constant weight, were broken into two halves across the longitudinal edges. All longitudinal edges were coated with a waterproof compound. Then, the end face (break) of the samples was placed in a specialized container filled with water to a level at which the end face of the sample was immersed in water by 5–10 mm. After 10 min of immersion, the samples were removed from the water, wiped with a damp cloth in the area of the break, and weighed. After weighing, the samples were again placed in the container and weighed again after 90 min. Capillary water absorption was determined by the formula:

(4) $$W_{k}=K\;\left(m_{2}-m_{1}\right)$$

where $m_{2}$ is the sample mass when saturated with water after immersion for 90 min (kg); $m_{1}$ is the sample mass when saturated with water after immersion for 10 min (kg); *K* = 69.93 1/(m^2^ × min^0.5^).

SEM analysis of the geopolymer paste was performed on a Tescan scanning electron microscope (Brno, Czech Republic) with an Inca Energy 450/XT electron probe microanalysis system.

## 3. Results

**Figure 4:**
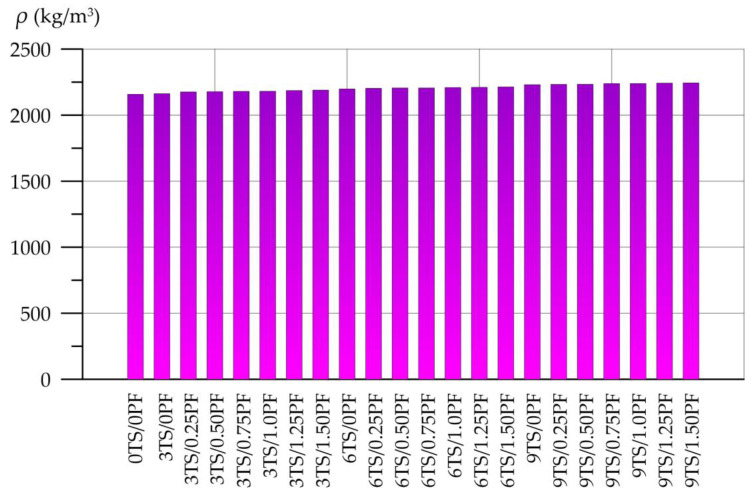
Geopolymer solution density dependence on TS and PF content.

**Figure 5:**
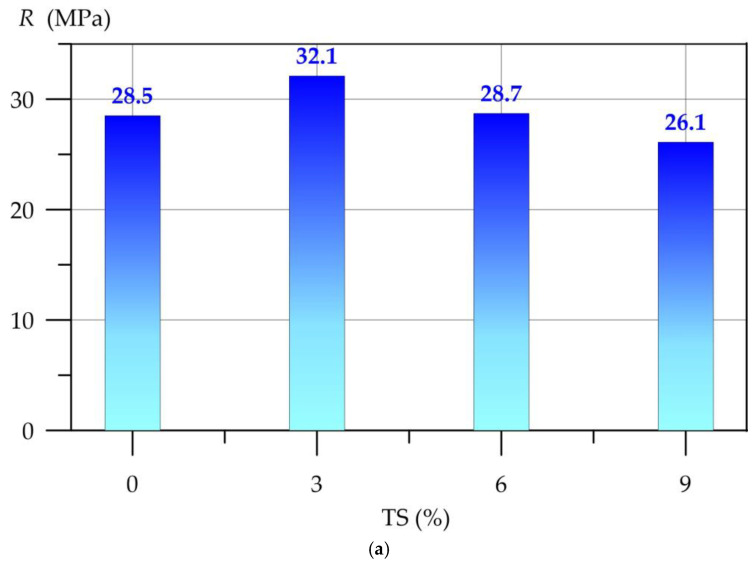

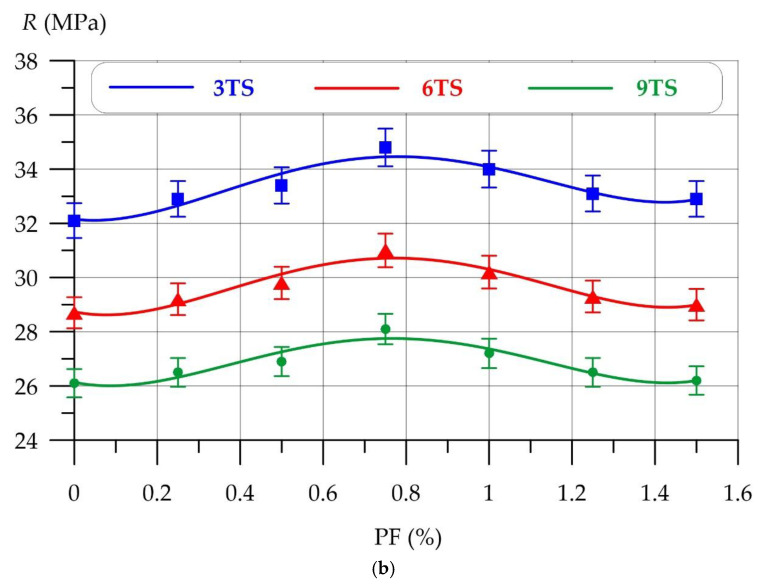
Compressive strength (*R*) of geopolymer mortar as a function of the amount of (**a**) TS, (**b**) TS and PF.

**Figure 6:**
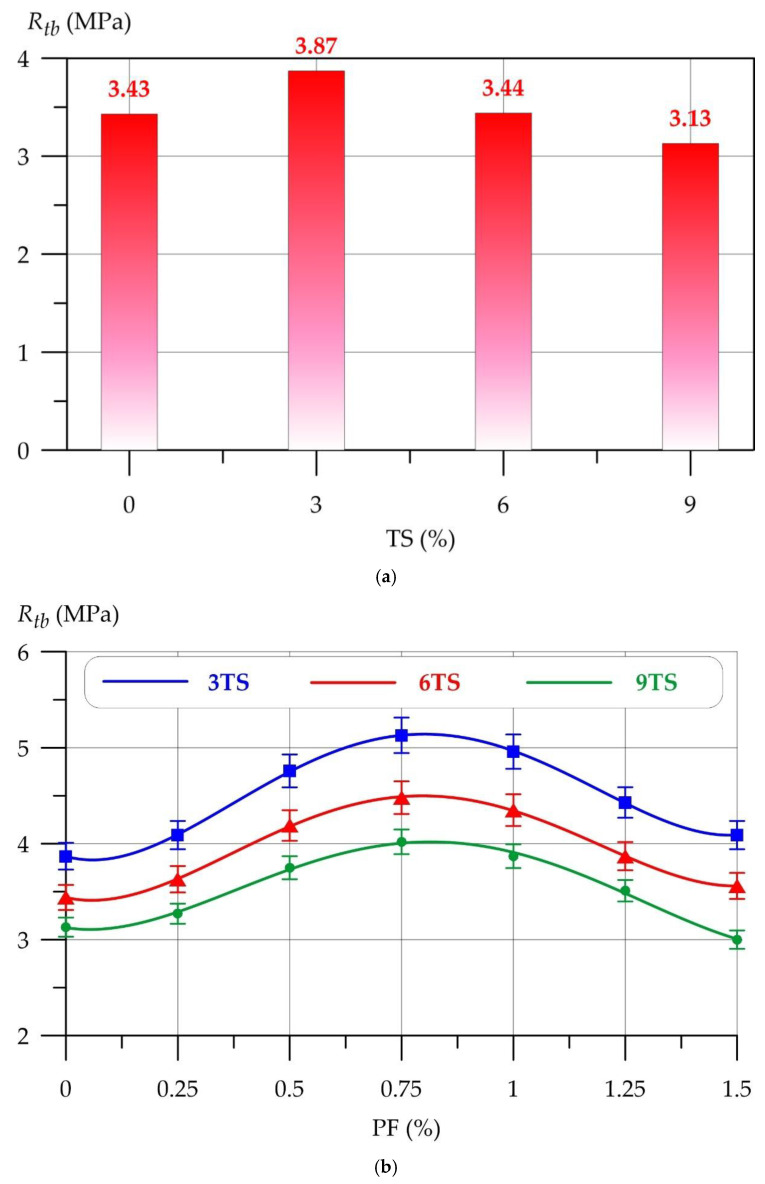
Geopolymer mortar flexural strength versus (**a**) TS, (**b**) TS and PF content.

**Figure 7:**
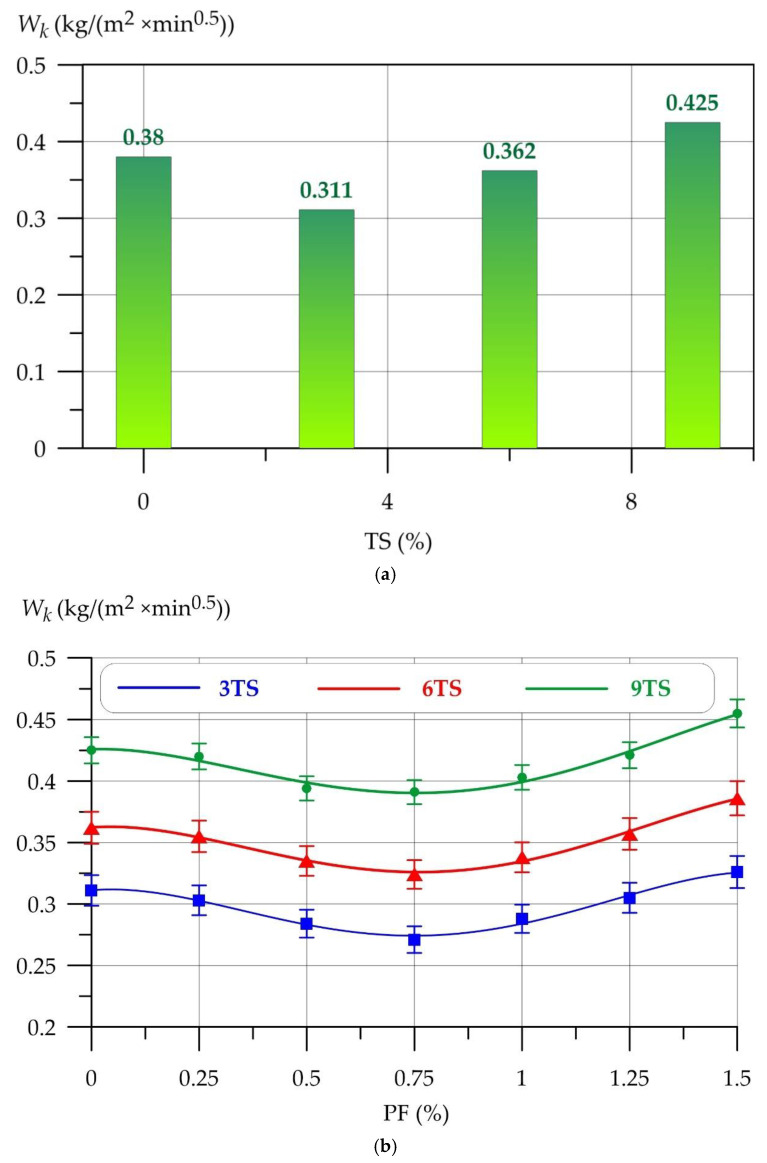
Dependences of capillary water absorption of geopolymer solution on the amount of (**a**) TS, (**b**) TS and PF.

The results of determining the density, compressive strength, flexural strength and capillary water absorption of geopolymer solutions are presented in Figure 4, Figure 5, Figure 6 and Figure 7. Figure 4 shows the dependence of the density of the geopolymer solution on the amount of TS and PF.

According to Figure 4, the density of geopolymer solutions modified with different amounts of TS and PF varies from 2158 kg/m^3^ to 2244 kg/m^3^. The difference compared to the control composition is no more than 4.0%. Stable small increases in density are observed as the dosages of the modifying additive and dispersed fiber increase.

Figure 5 shows the dependence of the compressive strength (*R*) of the geopolymer solution on the amount of TS and PF.

The dependences of the compressive strength (*R*) of the geopolymer mortar on the amount of PF at different values of TS are well approximated by 4th degree polynomials

(5) $$R^{3TS}\;=\;32.15-1.878\;x\;+\;21.65\;x^{2}-26.76\;x^{3}\;+\;8.921\;x^{4},\;\;\;\;\;R^{2}=0.916$$

(6) $$R^{6TS}\;=\;28.73-3.157\;x\;+\;23.69\;x^{2}-28.28\;x^{3}\;+\;9.309\;x^{4},\;\;\;\;\;R^{2}=0.938$$

(7) $$R^{9TS}\;=\;26.14-3.177\;x\;+\;21.44\;x^{2}-25.37\;x^{3}\;+\;8.339\;x^{4},\;\;\;\;\;R^{2}=0.891$$

where *R*^2^ is the coefficient of determination.

Figure 5a shows that the modification of geopolymer mortar with TS in an amount of 3% of the binder component weight has a positive effect on the compressive strength. Compared to the control composition, the increase in compressive strength was 12.6%. Then, with an increase in the TS content, the positive effect decreases sharply. At 6% TS, the compressive strength is comparable to the strength of the control composition. TS in an amount of 9% has a negative effect on the compressive strength, reducing it by 8.4%. The 3TS type composition has the best results compared to the 6TS and 9TS compositions. The inclusion of polypropylene fiber has a positive effect on the compressive strength. For all curves shown in Figure 5, a stable increase in compressive strength is observed at a PF content of up to and including 0.75%. The maximum values of compressive strength for compositions of the 3TS, 6TS and 9TS types at 0.75% PF were 34.8 Mpa, 31.0 Mpa and 28.1 Mpa, respectively. Further, with an increase in the amount of PF from 1% to 1.5%, the efficiency of dispersed reinforcement decreases. The values of increments depending on the combined modification of TS and PF are presented in Table 3.

Figure 6 shows the dependence of the flexural strength of the geopolymer solution on the amount of TS and PF.

The dependences of the flexural strength (*R*) of the geopolymer mortar on the amount of PF at different values of TS are well approximated by 4th degree polynomials

(8) $$R_{tb}^{3TS}=3.868-1.300\;x+12.16\;x^{2}-14.36\;x^{3}+4.596\;x^{4},\;\;\;\;\;R^{2}=0.999$$

(9) $$R_{tb}^{6TS}=3.439-1.043\;x+10.05\;x^{2}-11.90\;x^{3}+3.801\;x^{4},\;\;\;\;\;R^{2}=0.999$$

(10) $$R_{tb}^{9TS}=3.125-0.736\;x+7.570\;x^{2}-8.638\;x^{3}+2.589\;x^{4},\;\;\;\;\;R^{2}=0.996$$

As with the compressive strength, the 3TS composition has the highest flexural strength of 3.87 MPa with an increase of 12.8%. With 6% TS modification, the flexural strength is comparable to the strength of the control composition. 9% TS has a negative effect on the flexural strength. The introduction of PF in an amount of up to 0.75% has a positive effect on all types of compositions with 3%, 6% and 9% TS. For all curves shown in Figure 6b, a stable increase in flexural strength is observed at a PF modification level of up to and including 0.75%. The maximum flexural strengths for 3TS, 6TS and 9TS compositions at 0.75% were 5.13 MPa, 4.48 MPa and 4.02 MPa, respectively. Further, with an increase in the amount of PF from 1% to 1.5%, the efficiency of dispersed reinforcement decreases. The values of changes in the bending strength under the influence of the combined modification of TS and PF are presented in Table 4.

Further, Figure 7 shows the results of determining the capillary water absorption of the geopolymer solution modified with TS and PF.

The dependences of capillary water absorption (*W_k_*) of the geopolymer mortar on the amount of PF at different values of TS are well approximated by 4th degree polynomials

(11) $$W_{k}^{3TS}\;=\;0.311+0.030\;x-0.358\;x^{2}+0.444\;x^{3}-0.143\;x^{4},\;\;\;\;\;R^{2}=0.984$$

(12) $$W_{k}^{6TS}\;=\;0.362+0.026\;x-0.326\;x^{2}+0.392\;x^{3}-0.119\;x^{4},\;\;\;\;\;R^{2}=0.992$$

(13) $$W_{k}^{9TS}\;=\;0.426+0.011\;x-0.275\;x^{2}+0.338\;x^{3}-0.100\;x^{4},\;\;\;\;\;R^{2}=0.978$$

According to Figure 7a, the lowest value of capillary water absorption is shown by the 3TS type composition. The modification with 3% TS allowed to reduce capillary water absorption to 18.2% compared to the control composition of type 0TS. With the inclusion of 6% TS, capillary water absorption decreased to 0.362 kg/(m^2^ × min^0.5^) (4.7%). TS introduced into the geopolymer composite in the amount of 9% negatively affects capillary water absorption and increases it by 11.8%. PF has a positive effect on capillary water absorption for all compositions of type 3TS, 6TS and 9TS. The strength curves in Figure 7b show that PF in the range from 0.25% to 0.75% has a stable positive effect, with a peak best value at 0.75%. At this dosage, 3TS, 6TS and 9TS have the lowest capillary water absorption values of 0.271 kg/(m^2^ × min^0.5^), 0.324 kg/(m^2^ × min^0.5^) and 0.391 kg/(m^2^ × min^0.5^) respectively. The values of changes in the capillary water absorption of geopolymer mortars are presented in Table 5.

**Figure 8:**
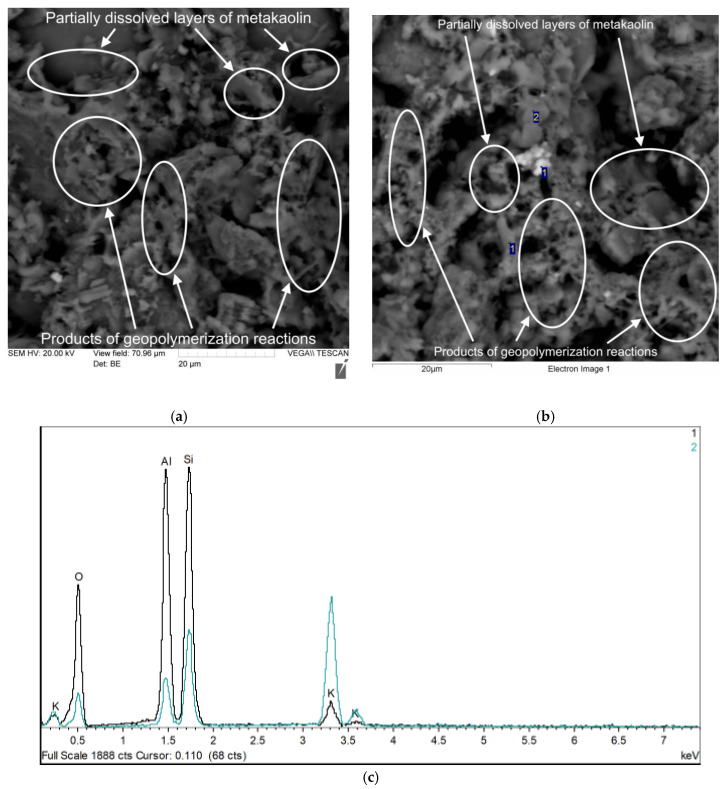
Geopolymer mortar sample of 0TS type composition: (**a**) microstructure with 2000× magnification; (**b**) EDX analysis (2000×); (**c**) EDX analysis result.

**Figure 9:**
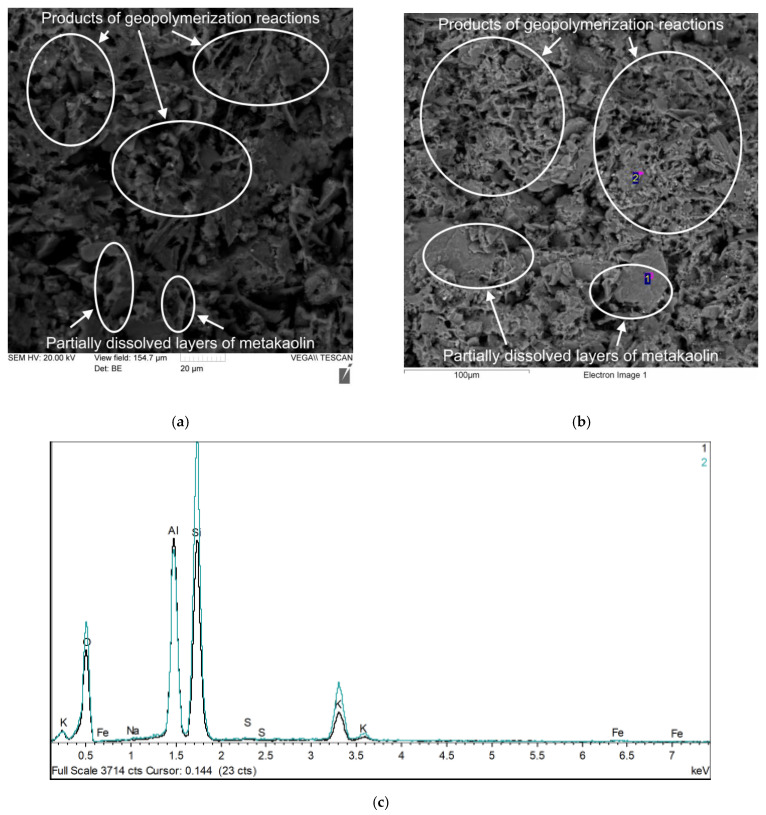
Sample of geopolymer concrete of 3TS type composition: (**a**) microstructure with 2000× magnification; (**b**) EDX analysis (1000×); (**c**) result of EDX analysis.

**Figure 10:**
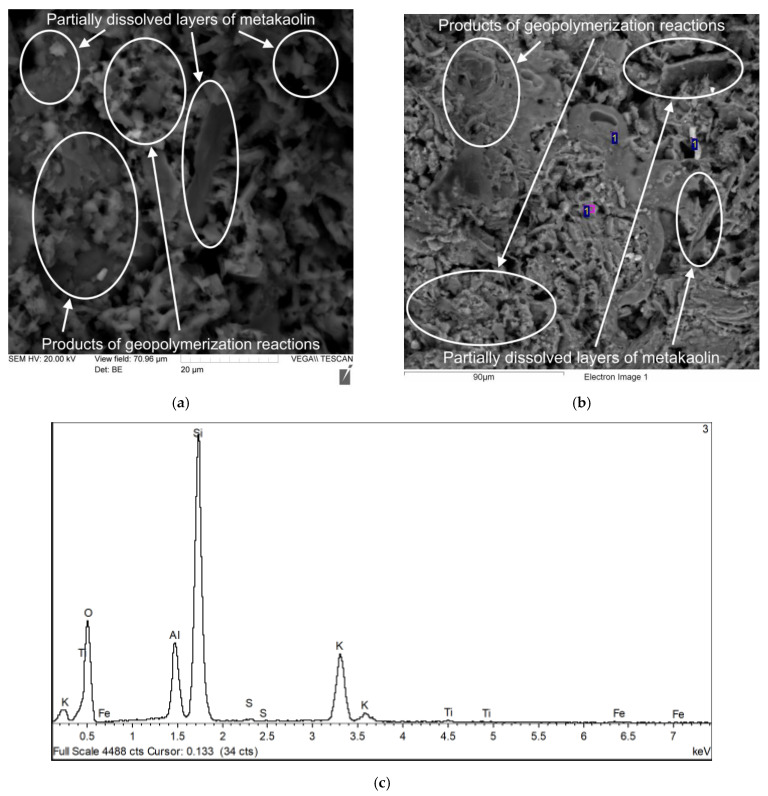
Sample of geopolymer concrete of 6TS type: (**a**) microstructure with 2000× magnification; (**b**) EDX analysis (700×); (**c**) result of EDX analysis.

**Figure 11:**
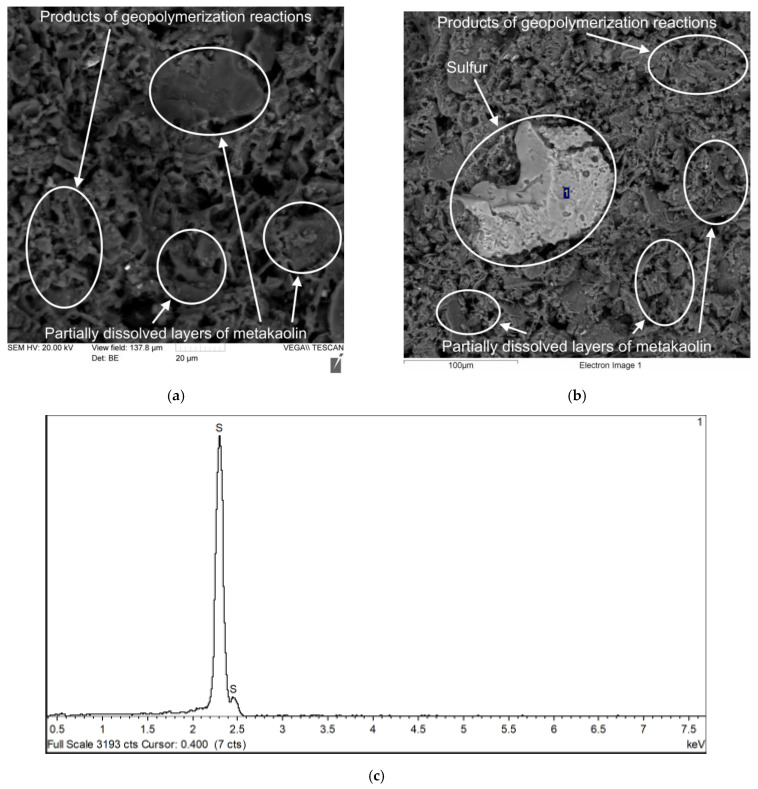
Sample of geopolymer concrete of type 9TS: (**a**) microstructure with 2000× magnification; (**b**) EDX analysis (500×); (**c**) result of EDX analysis.

Figure 8, Figure 9, Figure 10 and Figure 11 show photographs of the microstructure of the geopolymer matrix of compositions of the 0TS, 3TS, 6TS and 9TS types and EDX spectra characterizing the elemental composition. Figure 8 shows the microstructural and EDX analyses of the control composition of the geopolymer solution.

The structure of the geopolymer matrix in Figure 8a,b is represented by a homogeneous porous network formed by gel-like products of the geopolymerization reaction and partially dissolved metakaolin layers. Based on the results of the EDX analysis (Figure 8c), the following chemical elements were identified: Al, Si, O, K. Figure 9 shows the microstructural and EDX analyses of the 3TS geopolymer mortar composition.

The geopolymer matrix modified with 3% TS (Figure 9a) has a denser structural network formed by the products of the geopolymerization reaction and partially dissolved metakaolin layers in comparison with the geopolymer matrix of the control composition. Undissolved sulfur particles in the matrix structure were not detected according to the results of electron microscopy. The following chemical elements were identified according to the results of EDX analysis (Figure 9b,c): Al, Si, O, K, Fe, Na and S. Figure 10 shows the microstructural and EDX analyses of the 6TS geopolymer mortar composition.

The geopolymer matrix with 6% TS (Figure 10a) is characterized by an uneven porous network formed by gel-like products of the geopolymerization reaction and partially dissolved metakaolin layers, which are represented by plates. Undissolved sulfur particles are also present. According to the results of EDX analysis (Figure 10b,c), the following chemical elements were identified: Al, Si, O, K, Fe, S and Ti. Figure 11 shows the microstructural and EDX analyses of the 9TS geopolymer solution.

The geopolymer matrix with 9% TS, like the matrix with 6% TS, has a structure of an uneven porous network formed by gel-like products of the geopolymerization reaction and partially dissolved metakaolin layers, which are represented by plates (Figure 11a). A large accumulation of adhered and unreacted sulfur particles was recorded, which is confirmed by EDX analysis (Figure 11b,c) [53,54]. Thus, based on the results of the analysis of the microstructure of geopolymer matrices modified with TS additive in the range from 0% to 9% with a step of 3%, it was determined that the geopolymer matrix with 3% TS has the densest structure in comparison with the unmodified geopolymer matrix. Geopolymer matrices with excess TS content have a more porous structure, unreacted sulfur particles are identified and a large number of partially dissolved metakaolin layers are present [55,56].

## 4. Discussion

In general, based on the results of determining the density, compressive strength, flexural strength and capillary water absorption, the optimal ranges of the modifying additive and fibers were established. The introduction of TS in an amount of 3% is the best option and allows for an increase in strength properties up to 12.8% and a decrease in water absorption up to 18.2% compared to the control composition. The negative and positive effects of TS on the mechanical properties of the geopolymer are explained by different mechanisms of interaction of TS with the solution part of the geopolymer matrix [50,57]. With an optimal amount of TS up to 3%, it performs the function of a reactive component. TS is completely dissolved in the alkaline activator and has the best chemical stabilization in the structure of the geopolymer matrix, which is proven by the highest compressive and flexural strength. Excessive TS content leads to the fact that TS cannot fully enter into geopolymerization reactions. Unreacted sulfur particles remain in the structure of the geopolymer matrix. They have low strength properties and adhesive bonds at the interface with other components and are easily dissolved in water, which leads to the formation of additional stresses, cracks and voids in the matrix structure. All these factors lead to a decrease in strength properties [50]. The role of sulfur in geopolymers, either as a precursor or as a water-soluble filler, can be explained by comparing the S/Al molar ratio of the precursors and geopolymers using additional analyses. Sulfur reacts with aluminum ions from metakaolin and is incorporated into the microstructure of geopolymers. The crystalline phases of the resulting geopolymers are clearly changed after the addition of sulfur. SEM analysis shows that the microstructure of geopolymers became more dense and compact after the addition of sulfur. The fact that there is no unreacted sulfur in the microstructure of geopolymers although it was added to the precursors and dissolved in the alkaline solution is also consistent with the study [50,57]. Thus, sulfur can be initially used to improve the properties of geopolymers and facilitate the appearance of new phases and microstructural characteristics. The addition of sulfur to the alkaline solution at higher concentrations leads to the precipitation of elemental sulfur in the microstructure of the geopolymer. Sulfur becomes soluble in water, which leads to deterioration of the mechanical characteristics of the geopolymer [50,57]. PF acts as a dispersed reinforcement, the efficiency of which directly depends on the number of fibers and the degree and nature of their distribution throughout the structure of the geopolymer matrix. Thus, at 0.75% PF is uniformly distributed throughout the geopolymer matrix, additional macrostructural bonds are created, due to which the entire structure is compacted, and the strength of the entire geopolymer composite increases. PF compensates for part of the destructive load, and the distribution of deformation resistance forces is more uniform throughout the entire volume of the composite. The stress level in particularly dangerous and weak structural zones is reduced, and cracking is prevented [58,59,60]. It is worth noting that the results obtained from this study regarding modification with TS and PF additives are consistent and confirmed by a number of other scientific studies (Table 6 and Table 7). Table 6 presents typical examples of successful application of sulfur in geopolymer and low-cement composites.

Thus, the studies presented in Table 6 confirm the possibility of using various types of waste with a high TS content as part of the binder component or modifying additive in the composition of geopolymer composites. Optimally selected composition of aluminosilicate components and alkaline activator will allow obtaining sufficiently strong and stable composites with a dense structure, low shrinkage during drying and high resistance to aggressive effects [50,61,62,63,64].

Table 7 presents typical examples of the successful use of polypropylene fiber in geopolymer composites.

As can be seen from Table 7, dispersed reinforcement of various types of geopolymer composites with polypropylene fiber in an amount from 0.2% to 1.5% affects the properties of the geopolymer exclusively positively. The introduction of polypropylene fibers in an optimal amount into the composite significantly limits the propagation of cracks and increases its strength properties. The structural density of the composites and their resistance to aggressive chloride ions also increase [65,66,67,68,69,70,71]. It should be noted that the addition of sulphur and fibers may affect the rheology and workability of the fresh mix, and it is necessary to discuss the mixing parameters, consistency and necessary adjustments, such as the addition of water. It should be noted here that in each specific case, depending on, for example, the construction or production conditions, additional requirements for water content may arise. It is important to understand that the adjustment of any composite material by adding other components must be carried out in strict accordance with the requirements of regulatory and technical or technological documents. Due to the fact that the proposed composition is new and suitable for certain tasks, each adjustment of the proposed composition should be made after conducting pilot experimental studies. The addition of water or other components, as well as the required consistency, is directly dependent on the decision of the process engineer selecting the composition of the concrete mix and the technical specifications issued by the designer or the manufacturing company. In this regard, there is no universal formula regarding these components, and clarifications are necessary in each specific case. The results obtained in this study reflect the influence of the formulation parameters of the dosage of sulfur and polypropylene fiber and complement the theoretical and practical information of the global science on geopolymer composites [72]. It was found that, in an amount of up to 3%, TS acts as a reactive component in the geopolymer and completely dissolves in the structure of the geopolymer matrix, thereby strengthening it. A higher TS content has a negative effect on the properties of the geopolymer. This is due to the fact that sulfur particles cannot completely dissolve and remain in the matrix structure, which is confirmed by EDX analysis (Figure 10 and Figure 11). Dispersed PF reinforcement from 0.25% to 1.25% has a positive effect on the mechanical properties of the geopolymer. PF fibers, when uniformly distributed throughout the geopolymer matrix, have a reinforcing effect, binding all structural components of the geopolymer together. Macrostructural cells, “fiber-solution part” are created, and the entire structure of the matrix as a whole is strengthened [58,59,60,73].

The mechanisms of increasing strength when adding both TS and PF are manifested in a synergistic effect that has not been studied before. Technical sulfur forms a structural framework at the chemical level and binds the filler, forming a single matrix. Fiber contributes to increasing the tensile strength of the matrix due to dispersed reinforcement. The combination of two factors leads to a mutual effect of increasing strength at the micro- and macrostructural levels. The effect of technical sulfur is also manifested in the ordering of the geopolymer microstructure and the formation of fine porosity. Dispersed reinforcement of PF, as in the case of strength properties, has a positive effect on capillary water absorption for all compositions. The resulting geopolymer composites have sufficient compressive and bending strength for their use in construction practice both in construction site conditions and in plant conditions for the manufacture of various products [74,75,76,77]. The main advantage of the developed geopolymer composites is their environmental friendliness and the possibility of recycling technical sulfur, which is a by-product of gas processing and oil refineries. The developed geopolymer solutions with sulfur and fiber have sufficient strength properties, which determine their wide range of application. They can be used as plastering and masonry solutions for finishing the facades of buildings and structures for various purposes. These solutions can also be used to manufacture small-piece products, such as small wall blocks and steps. Under construction site conditions, the geopolymer solution can be used to manufacture various structural elements of buildings and structures. The main risks of this study include the poorly understood issues related to the use of sulfur-modified geopolymers in the long term under various operating conditions, in particular in an aggressive environment with a high content of chloride and sulfate ions. This fact will limit and restrain the possibility of using sulfur-modified geopolymer solution in real construction practice, especially under aggressive operating conditions. To eliminate the above risks, future studies are aimed at studying the durability properties of the developed geopolymers.

## 5. Conclusions

The possibility of using technical sulfur as a modifying additive in geopolymer technology has been studied. The optimal amount of technical sulfur and polypropylene fiber in the geopolymer composite has been determined.

(1) The possibility of using TS as a modifying additive in the manufacture of geopolymer composites has been proven. The maximum positive effect was recorded with a TS content of 3%. The increase in compressive strength was 12.6%, and flexural strength 12.8%. Capillary water absorption decreased by 18.2%. The optimal TS content, which allows obtaining a geopolymer without significant deterioration in properties, is up to 6%.
(2) The addition of PF additionally improves the properties of geopolymer solutions modified with TS. The maximum increases in strength properties were recorded for the composition with 0.75% PF and 3% TS. The increases in compressive and flexural strength were 8.4% and 32.6% compared to the 3TS type composition. Capillary water absorption decreased by 12.9%. The optimal PF content for this type of geopolymer composite ranged from 0.25% to 1.25%.
(3) Analysis of the microstructure of geopolymer matrices with different TS content shows that the best composition with 3% TS has the most homogeneous dense structure, represented by a network of gel-like products of the geopolymerization reaction and partially dissolved metakaolin layers, compared to the structure of the control composition. According to the results of EDX analysis of the composites, the following chemical elements were identified: Al, Si, O, K, Fe, Na and S. In the optimal amount of up to 3%, TS acts as a reactive component, completely dissolves in the alkaline activator and has better chemical stabilization in the structure of the geopolymer matrix.
(4) The most rational compositions of geopolymer mortar based on metakaolin and fly ash, modified with sulfur and polypropylene fiber, in terms of microstructure and properties, were obtained.
(5) Compared with traditional alkali-activated geopolymers, the resulting composite of carefully selected composition has better mechanical and physical characteristics and a more organized and ordered structure, and at the same time partially solves the problem of recycling accumulating sulfur waste.
(6) In the future, to confirm the effectiveness of the obtained composite in a wide range of applications, it is planned to conduct studies aimed at studying:
  - the durability of sulfur-modified geopolymer concretes, namely, assessing resistance to the effects of chloride and sulfate environments and studying the degradation mechanism of this type of geopolymer in an aggressive environment;
  - reaction products and hydration mechanisms using additional analyses (X-ray phase, thermogravimetric and mercury porosimetry);
  - the influence of curing conditions of the composite on its characteristics;
  - the influence of different ratios of binder components on the final properties and behavior of the geopolymer composite with sulfur.

The prospects for the development of this research are associated with further study of curing conditions (temperature, humidity) and the scope of application of the obtained composite not only in construction, but also in other industries.

## Figures and Tables

**Table 1:** Properties of precursors.

Metakaolin								
Mass fraction of silicon oxide SiO_2_ (%)						55.2		
Mass fraction of aluminum oxide Al_2_O_3_ (%)						42.4		
Mass fraction of iron oxide Fe_2_O_3_ (%)						0.8		
Moisture (%)						0.1		
LOI (%)						1.5		
FA								
SiO_2_ (%)	Al_2_O_3_ (%)	Fe_2_O_3_ (%)	СаО (%)	MgO (%)	TiO_2_ (%)	P_2_O_5_ (%)	SO_3_ (%)	LOI (%)
50.82	20.5	7.71	5.55	2.18	0.87	0.21	0.16	12.0
Potassium liquid glass								
Density at temperature (20 ± 0.5) °C (g/cm^3^)						1.41 ± 0.03		
Mass fraction of potassium oxide (K_2_O) (wt%)						10.8 ± 0.3		
Mass fraction of silicon dioxide (SiO_2_) (wt%)						23.8 ± 0.5		
Silicate modulus						2.2 ± 0.1		
QS								
Bulk density (kg/m^3^)						1360		
Apparent density (kg/m^3^)						2564		
The content of dust and clay particles (%)						0.07		
Content of clay in lumps (%)						0		
Fineness modulus						1.63		
TS								
Bulk density (kg/m^3^)						1338		
Sulfur mass fraction (%)						99.95		
Ash content (%)						0.02		
Mass fraction of organic substances (%)						0.01		
Mass fraction of acids in terms of sulfuric acid						0.002		
Mass fraction of water (%)						0.018		
PF								
Fiber diameter (µm)						12		
Fiber length (mm)						16–24		
Tensile strength (MPa)						320		
Density (g/cm^2^)						0.91		

**Table 2:** Compositions of geopolymer solutions.

Mixture Type	M (g)	FA (g)	K_2_O(SiO_2_)_n_ (g)	QS (g)	TS (g)	PF (g)
0TS/0PF	1080	120	1275	4050	0	0
3TS/0PF	1080	120	1275	4050	36	0
3TS/0.25PF	1080	120	1275	4050	36	3
3TS/0.50PF	1080	120	1275	4050	36	6
3TS/0.75PF	1080	120	1275	4050	36	9
3TS/1.0PF	1080	120	1275	4050	36	12
3TS/1.25PF	1080	120	1275	4050	36	15
3TS/1.50PF	1080	120	1275	4050	36	18
6TS/0PF	1080	120	1275	4050	72	0
6TS/0.25PF	1080	120	1275	4050	72	3
6TS/0.50PF	1080	120	1275	4050	72	6
6TS/0.75PF	1080	120	1275	4050	72	9
6TS/1.0PF	1080	120	1275	4050	72	12
6TS/1.25PF	1080	120	1275	4050	72	15
6TS/1.50PF	1080	120	1275	4050	72	18
9TS/0PF	1080	120	1275	4050	108	0
9TS/0.25PF	1080	120	1275	4050	108	3
9TS/0.50PF	1080	120	1275	4050	108	6
9TS/0.75PF	1080	120	1275	4050	108	9
9TS/1.0PF	1080	120	1275	4050	108	12
9TS/1.25PF	1080	120	1275	4050	108	15
9TS/1.50PF	1080	120	1275	4050	108	18

**Table 3:** Change in compressive strength of geopolymer mortar from the content of TS and PF.

TS (%)	PF (%)					
	0.25	0.50	0.75	1.0	1.25	1.5
	∆R (%)					
3	2.5	4.0	8.4	5.9	3.1	2.5
6	1.7	3.8	8.0	5.2	2.1	2.4
9	1.5	3.1	7.7	4.2	1.5	1.1

**Table 4:** Change in the bending strength of the geopolymer solution from the content of TS and PF.

TS (%)	PF (%)					
	0.25	0.50	0.75	1.0	1.25	1.5
	∆R_tb_ (%)					
3	5.7	23.0	32.6	28.9	14.5	5.7
6	5.5	21.8	30.2	26.5	12.5	3.5
9	4.5	19.8	28.4	23.6	12.1	−4.2

**Table 5:** Change in capillary water absorption of geopolymer mortar from TS and PF content.

TS (%)	PF (%)					
	0.25	0.50	0.75	1.0	1.25	1.5
	∆W_k_ (%)					
3	−2.6	−8.7	−12.9	−7.4	−1.9	4.8
6	−1.9	−7.5	−10.5	−6.6	−1.4	6.6
9	−1.2	−7.3	−8.0	−5.2	−0.9	7.1

**Table 6:** Analysis of the effect of TS modification on the properties of geopolymer.

Ref. Num.	Waste Type	Optimal Content (wt%)	Result
[50]	Sulfur waste from oil refineries	Up to 5%	Increase in compressive strength from 22.5 MPa to 29.9 MPa. Denser and more compact microstructure of the geopolymer
[61]	Sulfur-tailings	Main component of the binder	A geopolymer composite with compressive strength up to 32.2 MPa and high efficiency of heavy metal immobilization was obtained
[62]	SO_3_	4%	Increases in compressive and flexural strength by 17.91% and 15.56%
[63]	Desulfurization ashes	50%	The possibility of using desulphurization ash with high sulphur content as the main binder component in the manufacture of low-cement composites was proven
[64]	Waste from enrichment of sulfide iron ores	25–50%	Geopolymer composites with improved sulphate resistance and frost resistance were obtained

**Table 7:** Analysis of the effect of dispersed reinforcement with polypropylene fibers on the properties of geopolymer.

Ref. Num.	Fiber Type	Optimal Content (wt%)	Result
[65]	Polypropylene	0.2–0.4%	Increased flexural strength and higher resistance of geopolymer to chloride ion permeability
[66]		0.25–0.5%	Developed geopolymer concrete for 3D printing with improved productivity and required strength properties
[67]		0.5%	Improved mechanical properties and durability
[68]		1.0%	Increase in flexural strength by 65%
[69,70,71]		0.5–1.5%	Provided an increase in compressive and flexural strength of geopolymer composites

## Data Availability

The original contributions presented in the study are included in the article, further inquiries can be directed to the corresponding author.
